# Prediction of Protein-Destabilizing Polymorphisms by Manual Curation with Protein Structure

**DOI:** 10.1371/journal.pone.0050445

**Published:** 2012-11-26

**Authors:** Craig Alan Gough, Keiichi Homma, Yumi Yamaguchi-Kabata, Makoto K. Shimada, Ranajit Chakraborty, Yasuyuki Fujii, Hisakazu Iwama, Shinsei Minoshima, Shigetaka Sakamoto, Yoshiharu Sato, Yoshiyuki Suzuki, Masahito Tada-Umezaki, Ken Nishikawa, Tadashi Imanishi, Takashi Gojobori

**Affiliations:** 1 Integrated Database Group, Japan Biological Information Research Center, Tokyo, Japan; 2 Integrated Database Group, Biological Information Research Center, National Institute of Advanced Industrial Science and Technology, Tokyo, Japan; 3 Reconesis, Tokyo, Japan; 4 Laboratory for Gene-Product Informatics, Center for Information Biology and DNA Data Bank of Japan, National Institute of Genetics, Mishima, Japan; 5 Department of Bioinformatics, Maebashi Institute of Technology, Maebashi, Japan; 6 Laboratory for Medical Informatics, Center for Genomic Medicine, The Institute of Physical and Chemical Research (RIKEN), Yokohama, Japan; 7 Institute for Comprehensive Medical Science, Fujita Health University, Toyoake, Japan; 8 Department of Forensic and Investigative Genetics, Institute of Applied Genetics, University of North Texas Health Science Center, Fort Worth, Texas, United States of America; 9 Innovation Center Okayama for Nanobio-Targeted Therapy, Graduate Schools of Medicine, Dentistry and Pharmaceutical Sciences, Okayama University, Okayama, Japan; 10 Life Science Research Center, Kagawa University, Kagawa, Japan; 11 Department of Photomedical Genomics, Basic Medical Photonics Laboratory, Medical Photonics Research Center, Hamamatsu University School of Medicine, Hamamatsu, Japan; 12 Holonics Co., Numazu, Japan; 13 Department of Microbiology and Molecular Genetics, Graduate School of Pharmaceutical Sciences, Chiba University, Chiba, Japan; 14 Center for Information Biology and DNA Data Bank of Japan, National Institute of Genetics, Mishima, Japan; 15 Graduate School of Natural Sciences, Nagoya City University, Nagoya, Japan; 16 Biological Informatics Consortium, Tokyo, Japan; University of South Florida College of Medicine, United States of America

## Abstract

The relationship between sequence polymorphisms and human disease has been studied mostly in terms of effects of single nucleotide polymorphisms (SNPs) leading to single amino acid substitutions that change protein structure and function. However, less attention has been paid to more drastic sequence polymorphisms which cause premature termination of a protein’s sequence or large changes, insertions, or deletions in the sequence. We have analyzed a large set (n = 512) of insertions and deletions (indels) and single nucleotide polymorphisms causing premature termination of translation in disease-related genes. Prediction of protein-destabilization effects was performed by graphical presentation of the locations of polymorphisms in the protein structure, using the Genomes TO Protein (GTOP) database, and manual annotation with a set of specific criteria. Protein-destabilization was predicted for 44.4% of the nonsense SNPs, 32.4% of the frameshifting indels, and 9.1% of the non-frameshifting indels. A prediction of nonsense-mediated decay allowed to infer which truncated proteins would actually be translated as defective proteins. These cases included the proteins linked to diseases inherited dominantly, suggesting a relation between these diseases and toxic aggregation. Our approach would be useful in identifying potentially aggregation-inducing polymorphisms that may have pathological effects.

## Introduction

One of the most promising uses of the large amounts of genomic data now available is the prediction of which polymorphisms in human gene sequences cause phenotypic effects leading to human diseases. Many disease-related effects of polymorphisms involve effects on protein structure and/or function. Thus, accurate predictions of the effect of polymorphisms on the structure and function of protein gene products are essential for improvement of drug therapy, disease prevention based on lifestyle changes, and personalized medicine based on an individual’s particular protein variants. Because of this, emphasis has been placed on prediction of the effects of large numbers of nonsynonymous single nucleotide polymorphisms (nsSNPs), i.e., those leading to a single amino acid substitution, on protein structure and function [1], [2], [3]. These predictions have been based on consideration of nsSNPs’ effects on protein stability and structure, given the known three-dimensional (3D) protein structure or that of a homologue [1]–[8]. Some of these also included a consideration of sequence conservation among species at the relevant amino acid position [3], [5], [6], [7]. Many of these methods use rules, such as the “Sunyaev rules” [3], [6], [7], to automatically assign a probability that a given nsSNP will be structurally destabilizing. Some researchers have created databases with large numbers of nsSNP results [4], or servers applying the rules to user-supplied sequences [7], [9]. The rules and methods currently in use have success rates <80% for predicting protein-destabilizing effects, with large numbers of both false positives and false negatives.

However, the effects of polymorphisms that introduce modifications in protein sequence more drastic than a single amino acid substitution have not yet been studied or predicted. The public Web-accessible database dbSNP [10] (http://www.ncbi.nlm.nih.gov/projects/SNP/) contains large numbers of such polymorphisms, including nonsense SNPs that truncate a protein by introducing premature stop codons and indels. This latter class includes frameshifting indels that completely alter a protein’s sequence downstream from the indel location, and thus the sequence becomes out of frame (often introducing a premature stop codon also). The effects of these polymorphisms on protein structure and function, and their consequent relationships with human diseases, have not been examined in detail on a large scale.

These cases may appear trivial, since many severe truncations and/or frameshifts would cause such drastic changes as to be lethal, including creation of toxic aggregates. However, in both the public polymorphism database dbSNP [10] and in the Online Mendelian Inheritance in Man (OMIM) database of disease-related genes (http://www.ncbi.nlm.nih.gov/Omim), there are large numbers of such polymorphisms that are associated with disease, but are not lethal. This suggests that very significant changes in protein sequence, leading to proteins that are highly defective and perhaps even toxic, can be found in living individuals. This is consistent with the discovery that large numbers of drastically altered splice variants, with a high likelihood of being unstable, are found in human brain tissue [11]. Also, 256 human SNPs were found even at conserved spliced dinucleotides (GT-AG sites) that had been thought to be invariant because of their functional importance [12]. Furthermore, recent studies have described high frequencies of deletion polymorphisms in the human genome [13], [14]. Therefore these severe polymorphisms are quite common, indicating that the accurate identification of potentially protein-destabilizing cases is more important than previously supposed.

Our goal in this work was to find novel protein-destabilizing mutations/polymorphisms in genes already known and reported in OMIM, to be related to diseases. The pathological roles of the polymorphisms for which we have made predictions have not yet been reported; these polymorphisms have not yet been associated with diseases linked to the OMIM-reported genes in question. Therefore, we are making predictions of possible pathological effects of genetic polymorphisms by annotating protein-destabilizing effects of the known polymorphisms in dbSNP. To predict the presence or absence of deleterious effects, we used visual inspection of (both frameshifting and non-frameshifting) indels and nonsense SNPs aligned with protein 3D structures corresponding to each candidate disease-related cDNA, using either the actual experimental 3D structure (if known) or the structure of one or more other proteins homologous in sequence.

**Figure 1:**
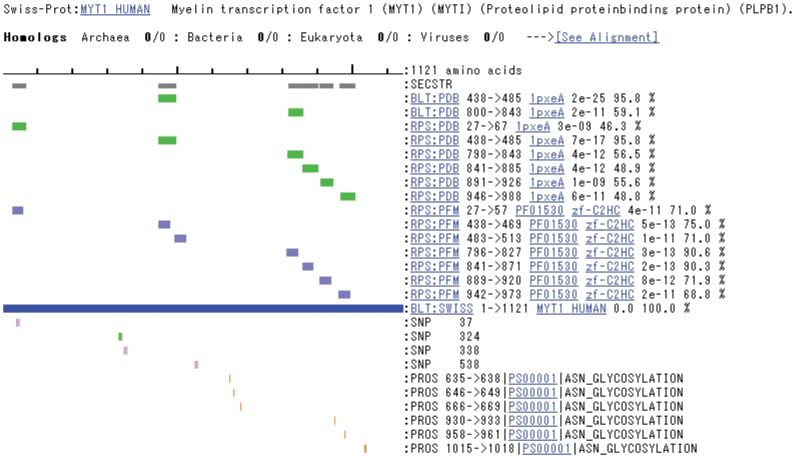
A sample view of the linear GTOP sequence alignment of the protein encoded by a cDNA with PDB structures. Multiple segments of the cDNA-encoded protein are each aligned with a small zinc protein sequence of known structure (PDB code 1pxe, chain A), whose sequence is represented by green bars. The small green and purple rectangles represent positions of polymorphisms.

## Methods

### Classification of Polymorphisms with Gene Structure

SNPs and indels in build 121 of dbSNP [10] were used in this study. We used the gene structure data of the H-Invitational full-length cDNA annotation project [15], [16]. For mapping cDNAs to the human genome, creating a correspondence between cDNAs and genomic loci and for selection of polymorphisms in cDNA, the result of cDNA mapping to the human genome (build 34) was used. The predicted ORF region for each cDNA was used to classify polymorphisms according to their possible effect on the ORF.

To analyze polymorphisms within predicted ORFs, nucleotide positions of polymorphisms (start and end positions) in the human genome sequences were converted into the nucleotide position in cDNA sequences, with the alignment of the cDNA sequence with the human genome sequence. When the cDNA sequence was corrected in terms of ORF prediction because of frameshifting and remaining introns, the nucleotide position of the polymorphism was modified based on addition or deletion of nucleotide sequences [17], [18].

### Alignment of Polymorphisms with GTOP

**Figure 2:**
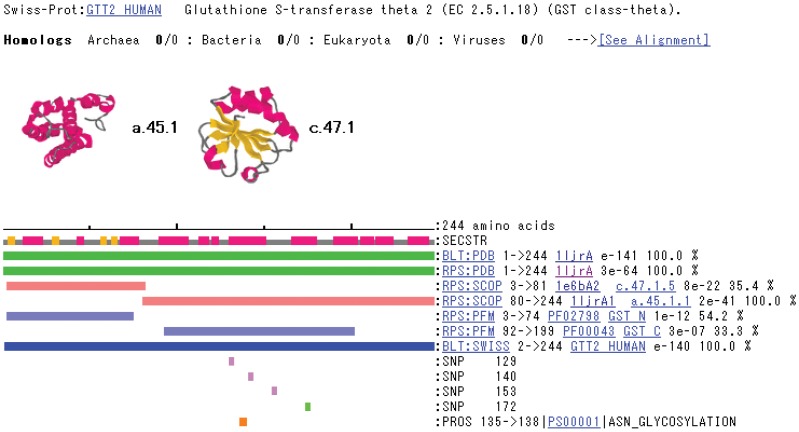
A sample view of the linear GTOP sequence alignment of the protein encoded by a cDNA with two SCOP domains. SCOP domains are represented as orange-pink bars and an aligned PDB structure as a green bar. In this case, the cDNA-encoded protein is entirely aligned with the crystal structure of its corresponding protein product. The green rectangle indicates a nonsense SNP. In this case, the polymorphism is completely within a SCOP domain and a PDB structure; in other cases, one or more polymorphisms may be between multiple structural domains to which the cDNA-encoded protein has been aligned, or upstream or downstream of all domains.

**Figure 3:**
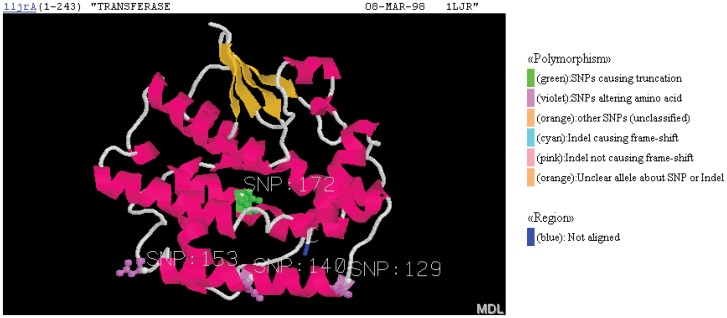
Three-dimensional representation of a SNP position within an aligned protein structure. The position of the nonsense SNP of Figure 2 is displayed within the corresponding GTOP CHIME image and PDB structure, showing the location of the nonsense SNP (green residue) in Chain A of PDB structure 1ljr. α-helices and β-sheets are shown in magenta and orange, respectively.

**Figure 4:**
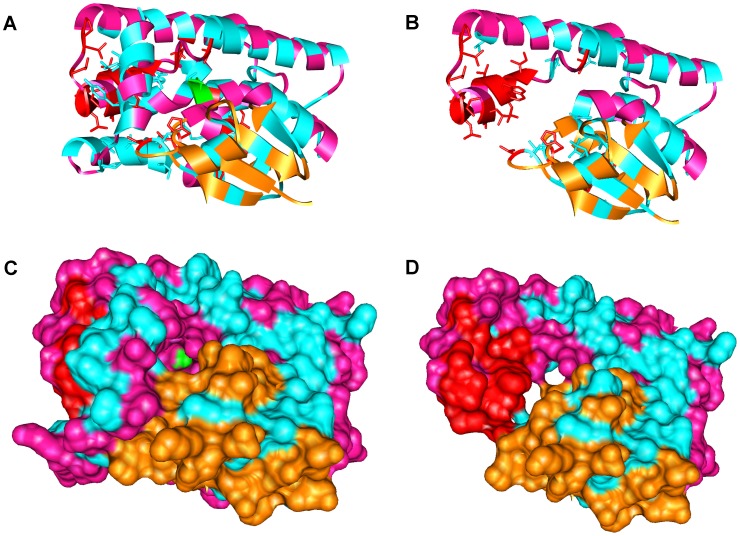
Example of truncation and hydrophobic core exposure caused by a nonsense SNP. The complete (A and C) and truncated (B and D) forms of the protein structure of Figure 3, shown in similar presentation of Figure 3A and 3B and with the molecular surface displayed (C and D). The green amino acid residue is the position of the nonsense SNP. Red color shows hydrophobic amino acids that are packed in the core of the protein in the complete protein but exposed in the truncated protein are colored. Cyan color shows other hydrophobic amino acids, including those that pack against the red portion in the complete protein. Other colors are as in Figure 3, with orange and magenta representing β-sheets and α-helices, respectively, and green representing the amino acid containing the SNP. In the truncated structure (D), there is a large area of exposed, normally buried hydrophobic surface (red) resulting from the truncation; this surface is buried under cyan and magenta regions (C).

Predicted ORF sequences for the cDNAs were aligned to Structural Classification of Proteins (SCOP) domains and experimentally determined structures in the Protein Data Bank [19] using BLAST and PSI-BLAST [20] within the Genomes TO Proteins (GTOP) database [21](http://spock.genes.nig.ac.jp/~genome/gtop.html). In the GTOP database, each alignment of polymorphism on the protein structure is incorporated into a web page using the CHIME plug-in (http://www.mdl.com) to display the protein domains with all SNPs and indels indicated and labeled as to position and type. The location of each polymorphism is indicated on a linear diagram showing the alignments of the sequence with different SCOP domains and PDB structures, as illustrated for sample cDNAs in Figures 1 and 2. A link to a CHIME 3D image of each structure to which all or part of the cDNA was aligned is included in this web page (Figure 3). All 3D images herein were prepared using the GTOP system and the CHIME plug-in, except for those of Figure 4, which were prepared with the UCSF Chimera package [22] from the Resource for Biocomputing, Visualization, and Informatics at the University of California, San Francisco (supported by NIH P41 RR-01081).

### Annotation of Nonsense SNPs and Indels with Protein Structure

The GTOP web browser displays were used to manually annotate each indel and nonsense SNP for its potential protein-destabilizing effects, considering the presence or absence of protein structure defects according to the following criteria:

- Nonsense SNPs and frameshifting indels (often causing premature termination) occurring in linker regions between protein structural domains were annotated as “possibly not protein-destabilizing” because at least one complete structural domain would remain upstream of the SNP or indel (e.g., green rectangle in Figure 1). If it were located downstream of all domains, then it would leave all domains intact and would be annotated as “not protein-destabilizing.”
- Nonsense SNPs and frameshifting indels occurring within a structural domain were annotated as “possibly protein-destabilizing” if it was evident from visual inspection that the hydrophobic core of the domain was exposed, likely leading to aggregation, a consequent loss of soluble protein and possible toxicity of the aggregated protein (see the more detailed discussion of this below).
- Nonsense SNPs and frameshifting indels occurring upstream from all structural domains were annotated as “possibly protein-destabilizing” because the result would be the loss of all structural domains.
- Non-frameshifting indels within a linker region (not within a structural domain) were annotated as “possibly not protein-destabilizing” since they would have no effect on the structure of any domain.
- Non-frameshifting indels within a structural domain were annotated visually on a case-by-case basis, depending upon the length of the indel and its location on the surface or within the core of the domain.
- Ambiguous cases, those cases for which it was not possible to determine whether or not a particular polymorphism was protein-destabilizing using the above criteria, were annotated as “hold (ambiguous).”

Figures 2 and 3 are examples showing that the ability to visualize a truncating polymorphism’s position within a 3D structure is useful to determine whether that polymorphism has a protein-destabilizing effect due to exposure of hydrophobic core. The 3D image in Figure 3 shows that the nonsense SNP would cause a truncation within a packed bundle of α-helices, exposing to solvent much of the surface area of this hydrophobic core that was previously packed against other hydrophobic residues and involved in stabilization of the structure.

To illustrate this core exposure effect more clearly, Figure 4 shows the protein from Figures 2 and 3 before and after a truncation caused by a nonsense SNP. A large amount of surface area comprised of hydrophobic side chains, which was formerly buried in the protein and contributed to its stability by packing against other hydrophobic amino acids, is now exposed. This newly exposed surface area on protein monomers can now pack against part of the same area on other monomers, leading to formation of pathological aggregated multimers, which can be highly toxic.

### Prediction of Nonsense-mediated Decay

One additional criterion was used to predict polymorphisms that are protein-destabilizing, but only in a recessively transmitted manner: Any polymorphism predicted to be subject to nonsense-mediated decay (NMD) [23], [24], [25]was considered protein-destabilizing in a recessive (homozygous) sense, whether or not it would be considered protein-destabilizing according to the criteria described above. NMD prevents translation of proteins with premature nonsense codons via direct decay of those mRNAs, if the nonsense codon is located more than 50 bp upstream of the end of the penultimate exon [24], [25]. This additional criterion using NMD served to slightly reduce the number of ambiguous cases (annotated as “hold”) and the number of “not protein-destabilizing” cases, since some of these could be re-classified as recessively pathological just because of the position of a premature termination. However, truncated proteins not subject to NMD could in theory be toxic, if the truncation is within a domain and exposes enough hydrophobic surface area to induce aggregation. If this were a cause of disease, the effect would be expected to be dominant, and would thus occur even at heterozygous state with the normal allele. To investigate the ability of our method to predict cases in which disease is caused by truncation-induced aggregation due to a nonsense codon within a structural domain, we analyzed all of the polymorphisms leading to a premature termination to examine which of them are likely to be subject to NMD [17].

## Results and Discussion

### Annotation Statistics

In total, 182 polymorphisms were predicted to be structurally destabilizing by our manual annotation (Table 1). Protein-destabilization was predicted for 44.4% (107/241) of the nonsense SNPs and 32.4% (70/216) of the frameshifting indels. In contrast, most of the non-frameshifting indels (83.6%) were predicted to be not protein-destabilizing, as they occurred outside of SCOP or PDB domains, in linker regions between domains or to the N- or C-terminal side of all domains.

**Table 1:** Number of each type of polymorphism with a given effect prediction.

Type of polymorphism	Structurally destabilizing			Hold (ambiguous)^c^	Total
	Yes		No		
	Possible recessive effects^a^	Possible dominant effects^b^			
Nonsense SNP	68	39	98	36	241
Frameshifting indel	32	38	96	50	216
Non-frameshifting indel	5	0	46	4	55
All types	105	77	240	90	512

a A typical example is loss of function.

b A typical example is aggregation.

c In addition to this statistics, there were 53 ambiguous cases because of lack of homologous 3D structure and 37 ambiguous cases for other reasons.

Although higher proportions of “structurally destabilizing” were observed for nonsense polymorphisms and frameshifts than non-frameshifting-indels, a large number of the predictions for the two cases fell into another class, “not protein-destabilizing” when a polymorphism left at least one remaining *intact* structural domain, and no partial domains with exposed hydrophobic cores. If a polymorphism deletes one or more structural domains containing residues important for global protein folding, for enzymatic catalysis, or for binding of ligands or protein interaction partners, then the polymorphism may actually be severely deleterious and cause disease due to decrease or absence of function. These cases should be subjected to further analysis to reduce the number of false negatives, using known functional (catalytic, ligand binding, protein interaction partner binding) and/or structurally important residues in databases such as Swiss-Prot [26] (http://www.expasy.org/sprot).

The presence of many probably protein-destabilizing, but not lethal, truncations and frameshifts in human populations is quite striking, given that the dramatic changes in the relevant proteins can be considered protein-destabilizing even using the conservative criteria of this work. Those polymorphisms classified as protein-destabilizing fall into two categories: the ones that cause a possible recessive effect due to simple absence of the protein in question (loss of function which could be compensated for in the heterozygous case); and those that cause a possible dominant effect, because of toxic effects such as aggregation that lead to a disease phenotype even in the presence of one wild type copy. In our annotation, 16% (39/241) of the total nonsense SNPs, and 36% (39/107) of the protein-destabilizing nonsense SNPs, are classified as possibly pathological in a dominant/heterozygous sense because they are likely misfolded and subject to aggregation, and are probably translated because they are not predicted to be subject to NMD (see the more extensive discussion below of NMD). Similarly, 18% (38/216) of the total frameshifting indels, and 54% (38/70) of the protein-destabilizing frameshifting indels are possibly pathological in a dominant sense. This suggests that very drastic changes in protein sequence, leading to highly defective proteins, can be found in human populations. As mentioned in the Introduction, this is consistent with the large numbers of drastically altered splice variants found in human populations, of which 83% were likely to be translated because they did not meet the criteria for NMD [11].

The number of ambiguous polymorphisms of all types whose potential protein-destabilizing could not be determined, classified as “hold,” comprises 18% of the total (90/512). We annotated a small number of cases, 7% of the total annotated polymorphisms (37/512) and as “hold,” due to uncertainty regarding the aggregation potential of a protein truncated near the N- or C-terminal of a SCOP or PDB domain.

The revealing statistic about the power of our prediction and annotation method is that 59% (53/90) of the ambiguous cases (10% of the total annotated polymorphisms, or 53/512) were sequences for which no 3D structure with sufficient homology exists. Only 7% of polymorphisms were not classifiable as protein-destabilizing or not protein-stabilizing due to ambiguities in our criteria and protocols. In the cases where no published 3D structure exists, our method itself does not break down and lead to ambiguity; rather, it is impossible to locate the polymorphism relative to a structural domain if there are no appropriate PDB or SCOP domains.

As a result of the NMD analysis, several polymorphisms that would otherwise be classified as “not protein-destabilizing,” because they do leave at least one intact structural domain remaining are classified as structurally-destabilizing. In most of these cases, the nonsense occurs downstream of all structural domains, suggesting no structure-affecting effect, but the prediction of NMD occurring suggests that the termination causes loss of functional protein because NMD prevents translation.

Table 1 shows that 39 of the nonsense SNPs, or 36% of the protein-destabilizing SNPs, are possibly pathological in a dominant sense, because truncation well within a structural domain and the predicted absence of NMD indicate the potential for aggregation due to exposed hydrophobic surface area that would normally be within the hydrophobic core of the protein. Similarly, 38 of the indels, or 51% of the total protein-destabilizing indels, are possibly pathological in a dominant sense because of potential hydrophobic core exposure and absence of NMD. The total number of potential aggregation-prone polymorphisms is therefore 77 out of a total of 512. Thus, 15% of the known nonsense SNPs and indels found in this dataset in known disease-related genes, and obviously non-lethal, are of such a drastic nature that they could lead to toxic protein aggregates.

### Predicted Aggregation and its Relationship to Diseases

To investigate the possibility of aggregation being a cause of disease in the cases of some of the truncating polymorphisms, we examined the nature of the diseases reportedly linked by OMIM to the genes displaying the 39 nonsense SNPs and 38 indels (all frameshifting) predicted to be possibly pathological in a dominant sense. Since the formation of toxic aggregated protein is one possible reason (but not the only one; see the discussion below) for dominant inheritance, Table 2 lists the genes containing predicted aggregation-prone premature terminations not subject to NMD which are reported in OMIM to be inherited in an autosomal dominant fashion or for which the mode of inheritance is unclear, i.e., not conclusively recessive.

**Table 2:** Diseases transmitted in a dominant fashion and associated with aggregation-prone polymorphisms that are not subject to NMD.

Protein product	Nonsense polymorphisms^a^	Indels^a^	Dominantly transmitted diseases^c^
Paraoxonase 1	3	0	Coronary artery disease, susceptibility to; coronary artery spasms, susceptibility to (168820)
Pulmonary surfactant-associated protein A	1	0	Idiopathic pulmonary fibrosis (178500)
Carboxylesterase 1	1	0	Non-Hodgkin lymphoma (605027); B-cell chronic lymphocytic leukemia (151400)
Hemicentin (fibulin 6)	1	0	Age-related macular degeneration, age-related, 1 (608548)
Cardiac myosin-binding protein C	1	0	Familial hypertrophic cardiomyopathy 4 (115197); dilated cardiomyopathy 1A(115200)
Retinoblastoma-associated protein	0	1^b^	Retinoblastoma; retinoblastoma, incomplete penetrance type; osteosarcoma, retinoblastoma-related; pineoblastoma (180200); small cell cancer of the lung (182280); bladder cancer (109800)

a The numbers of nonsense SNPs and indels that were annotated in this study are shown.

b This is a frameshifting indel.

c Disease information from OMIM is shown (MIM number in parentheses).

It is intriguing that there is a correlation between severe protein defects (nonsense SNPs within structural domains) and particular types of diseases. For example, as described in OMIM, the relationship among the paraoxonase 1 gene, its polymorphisms, and coronary artery disease and coronary artery spasm is known but poorly understood mechanistically. The relation between particular nonsense SNPs and coronary artery disease and coronary artery spasm is documented but is not well understood. It is unknown whether increased risk of these diseases is related to the SNPs causing decreased or increased enzymatic activity, to altered expression of this gene, or to other reasons. As the inheritance is autosomal dominant, a simple deficiency of functional protein is not the cause. Our annotation results on three nonsense SNPs in this protein, and the predicted absence of NMD, suggest that formation of toxic aggregate could be related to these diseases.

There are at least four other examples of a correlation between an intra-domain truncation and a disease is a nonsense SNP. The first example is a nonsense SNP in the gene for pulmonary surfactant-associated protein A1, which is related to idiopathic pulmonary fibrosis. A report that a difference in aggregation tendency due to the R219W nsSNP may be related to the disease in question [27] suggests that the probable higher aggregation tendency due to an intra-domain truncation has similar effects. Moreover, the inheritance is autosomal dominant, indicating that a simple deficiency of functional protein is not the cause.

The second example is that a likely protein-destabilizing intra-domain truncation also occurs in carboxylesterase 1 (CES1), an increased deficiency of which is seen in non-Hodgkin lymphoma and B-cell chronic lymphocytic leukemia. Although this implies a simple deficiency, the inheritance is autosomal dominant, suggesting a more complex relationship possibly involving both aggregation and toxicity.

The third example is cardiac myosin-binding protein C, with a probably deleterious nonsense SNP within a structural domain, exposing hydrophobic side chains of a β-sheet. Polymorphisms (mostly nsSNPs) in this sarcomeric protein are associated with familial hypertrophic cardiomyopathy [28]and dilated cardiomyopathy [29], and are associated with these diseases even in the heterozygous case with the normal allele, showing dominant negative transmission.

The last example is a nonsense SNP removing about two-thirds of a hydrophobically packed domain mostly comprised of β-sheets, in hemicentin. An nsSNP in hemicentin is associated with age-related macular degeneration, and is transmitted in a dominant negative fashion [30], with the reason for this as yet unknown. Thus, this may be a candidate for aggregation-caused toxicity. Interestingly this is a disease associated with aging, and protein aggregation is believed to induce aging-related diseases due to “protein aging” that occurs more readily in some protein variants than in others [31].

It is possible that a simple loss-of-function mutation can lead to dominant negative inheritance; this occurs in the case of complex pathways, such as those involved in transcription factor regulation [32], [33], [34]. Alternatively, binding of defective protein to a protein interaction partner or a normal molecule of the same protein to form an oligomer could cause a defective oligomer. For example, this is believed to account for disease phenotypes caused by insulin receptor polymorphisms heterozygous with the wild type: the tyrosine kinase domain of the receptor requires two normal β-chains, and the combination of a normal and a mutant β-chain may have reduced functionality [35]. The same mechanism may be involved in the deleterious effects of polymorphisms on cardiac myosin-binding protein C, which binds other proteins in the sarcomere. In the latter case, it is fascinating that such a large disruption of a structural domain involved in binding other proteins is seen in human populations rather than being lethal in the developmental stage. Thus, in some cases, a dominant pattern of inheritance may not be indicative of aggregation-linked toxicity but only of loss or decrease of function. For instance, a truncated protein may still be capable of binding wild type monomers to form an oligomer with decreased or no function.

In other cases, a dominant/heterozygous effect may be due to toxicity of a misfolded protein in solution, rather than in the aggregated form. For example, though polyglutamine expansions are often the cause of protein aggregation, in ataxin-1 and the disease spinocerebellar ataxia type 1 they have been linked to a gain of function of a misfolded protein in solution, causing the protein to alter signal transduction pathways [36], [37]. Similarly in some of the cases of severe protein truncation, this type of gain of function may explain the autosomal dominant transmission. If this type of phenomenon rather than aggregation is the cause of the disease phenotype, may act as a basis for understanding the pathways involved by analysis of the exact structural defects, observable in the GTOP alignment that results from truncation of the protein in question.

The sole indel-containing gene linked to dominantly transmitted diseases in Table 2 is that for retinoblastoma-associated protein, linked to retinoblastoma, lung cancer, malignancies of bone and the pineal gland, and bladder cancer. These diseases are already linked to some polymorphisms resulting in severely defective proteins, such as nonsense SNPs and frameshifting indels (linked to retinoblastoma and the other malignancies) [38], [39], [40] and polymorphisms leading to loss of a splice acceptor site and consequent abnormal splicing (linked to bladder cancer) [36]. Thus, this gene and its associated diseases are already correlated with drastic changes in the translated protein such as those that would result from the frameshifting indel for which we have predicted a protein-destabilizing effect.

### Conclusions

Our approach for annotation has the following advantages: (1) predictions of deleterious effects are unlikely to be false positives, given the severe changes in protein structure involved; (2) low ambiguity, even though the manual annotation is generally subjective. Thus, we can confidently make disease-related high-probability predictions of deleterious effects, although we may miss some deleterious polymorphisms.

Using this method for annotation of disease-related indels and nonsense SNPs would provide the type and position of each polymorphism quickly and easily within a web browser and enable researchers to choose interesting cases for researchers. This method may also be used to find cases of pathological polymorphisms in candidate disease genes identified using other criteria. The presence of severe protein defects predicted to be deleterious would support the identification of a gene as being potentially related to the etiology of a disease.
